# Bioactivity-Guided Isolation of Antimicrobial and Antioxidant Metabolites from the Mushroom *Tapinella atrotomentosa*

**DOI:** 10.3390/molecules23051082

**Published:** 2018-05-04

**Authors:** Zoltán Béni, Miklós Dékány, Bernadett Kovács, Boglárka Csupor-Löffler, Zoltán Péter Zomborszki, Erika Kerekes, András Szekeres, Edit Urbán, Judit Hohmann, Attila Ványolós

**Affiliations:** 1 Spectroscopic Research, Gedeon Richter Plc., Gyömrői út 19-21, H-1103 Budapest, Hungary; z.beni@richter.hu (Z.B.); M.Dekany@richter.hu (M.D.); 2 Department of Pharmacognosy, University of Szeged, Eötvös u. 6, H-6720 Szeged, Hungary; kovacs.bernadett@pharmacognosy.hu (B.K.); csupor.boglarka@pharmacognosy.hu (B.C.-L.); zombozope@pharmacognosy.hu (Z.P.Z.); 3 Department of Microbiology, University of Szeged, Közép fasor 52, H-6726 Szeged, Hungary; kerekeserika88@gmail.com (E.K.); andras.j.szekeres@gmail.com (A.S.); 4 Institute of Clinical Microbiology, University of Szeged, Semmelweis u. 6, H- 6725 Szeged Hungary; urban.edit@med.u-szeged.hu; 5 Interdisciplinary Centre for Natural Products, University of Szeged, Eötvös u. 6, H-6720 Szeged, Hungary

**Keywords:** *Tapinella atrotomentosa*, terphenyl quinones, antibacterial, antioxidant, multiresistant *Acinetobacter baumannii*, ESBL *Escherichia coli*

## Abstract

Bioassay-guided fractionation of the chloroform extract of *Tapinella atrotomentosa* led to the isolation of four secondary metabolites **1**–**4**. Two of the compounds are lactones—osmundalactone (**1**) and 5-hydroxy-hex-2-en-4-olide (**2**)—while **3** and **4** were identified as terphenyl quinones, spiromentins C and B, respectively. The structures of the compounds were established on the basis of NMR and MS spectroscopic analysis. The isolated fungal metabolites were evaluated for their antibacterial activities against several Gram-positive and negative bacteria. In addition, their synergistic effect with cefuroxime against methicillin-resistant *Staphylococcus aureus* (MRSA) was also evaluated. Compounds **1**–**3** proved to possess significant antibacterial activity against multiresistant *Acinetobacter baumannii* and extended-spectrum β-lactamase (ESBL)-producing *Escherichia coli*. The investigation of the antioxidant effect of the isolated compounds in DPPH and ORAC assays revealed that spiromentins C (**3**) and B (**4**) have remarkable antioxidant activity.

## 1. Introduction

Fungi and primarily Basidiomycota mushrooms are recognized as valuable sources of natural products with a great structural diversity, including cyclic peptides, steroids, sesquiterpenes and polysaccharides. They are known to exhibit various beneficial pharmacological properties such as antibacterial, immunmodulatory, hypocholesterolemic and antioxidant activities with a considerable therapeutic potential [1].

In our search for higher mushrooms with antibacterial activity we previously demonstrated that several species native to Hungary exerted notable activity on Gram-positive and negative bacteria. Among these the extracts of *Tapinella atrotomentosa* (Batsch) Šutara revealed not only a broad-spectrum antimicrobial activity on Gram-positive and negative pathogens, but demonstrated also a significant inhibitory activity against resistant bacterial strains [2]. *T. atrotomentosa*—velvet rollrim by its vernacular name—is a wood-rotting mushroom belonging to the Tapinellaceae family. It has a global distribution, being a quite common species in Europe, Asia and North-America, occurring on the roots and stumps of dead *Pinus* species. Although it was once consumed in some regions of Eastern Europe, this species is now considered inedible due to its bitter taste.

Previous studies have revealed the presence of a variety of compounds in this species. Gaylord et al. identified diphenyl-substituted tetronic acid pigments from cultures of *T. atrotomentosa*, namely xerocomic acid and atromentic acid [3]. The species produces orange-yellow flavomentin and violet spiromentin pigments, which possess terphenylquinone structures [4]. Atromentin, the 4,4-dihydroxy analogue of polyporic acid, accounts for the reddish-brown color of the external parts of *T. atrotomentosa* [5]. Leucomentins, the colorless precursors of atromentin also occur in the species [6]. Further to these, the species is known to biosynthesize several lactone type compounds including osmundalactone and bis-osmundalactone [7,8]. Velvet rollrim belongs to the few mushroom species containing rare ergostane-type ecdysteroids (e.g., paxillosterone and atrotosterones A–C) [9].

Based on the promising results of our previous antimicrobial screening study *T. atrotomentosa* has been selected for further detailed mycochemical experiments to determine its secondary metabolites responsible for the observed antibacterial properties. The present study reports on the major outcomes of the bioactivity-guided isolation of the extract of *T. atrotomentosa* and the pharmacological evaluation of the isolated compounds.

## 2. Results and Discussion

In the last years bacterial resistance to first-choice antibiotics have become a major problem with global impact on human population. The association between multiresistant microorganisms and nosocomial infections places a huge burden on health care systems in many countries around the world. This critical situation is even worsened by the fact that in the past decades the antimicrobial research was a fairly neglected area. In this perspective, there is an urgent need for more intense search to develop new anti-infective agents to enter the antimicrobial therapy. The contribution of mushroom metabolites to antibacterial therapy is low, but with a large unexplored potential. Most of the studies dealing with the antimicrobial properties of higher fungi report on the activity of mushroom’s extracts of different polarities without identifying the compounds responsible for the observed activity. Nevertheless some antibacterial fungal metabolites were isolated e.g., anthraquinones from *Cortinarius basirubescens* [10], sesquiterpenes from the edible and medicinal species of *Flammulina velutipes* [11], and prenylphenyl derivatives from *Albatrellus flettii* [12].

**Figure 1:**
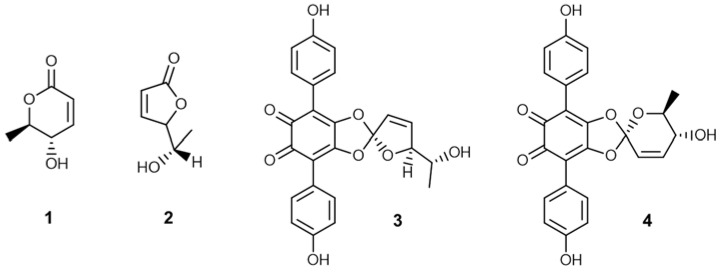
Structures of compounds **1**–**4** isolated from *T. atrotomentosa*.

In the framework of our project to investigate mushrooms native to Hungary for their potential pharmacological benefits we found that extracts of *T. atrotomentosa* exerted a broad spectrum antimicrobial activity on several bacterial strains. Detailed mycochemical experiments were carried out to identify the major compounds with antibacterial potential. The collected mushroom material was extracted with methanol on room temperature. The crude extract was subjected to solvent-solvent partition with *n*-hexane and then chloroform. The chloroform extract was separated in multiple steps using flash chromatography on normal phase. The final purification carried out by normal phase HPLC resulted in compounds **1**–**4**. The isolated metabolites were subjected to NMR and MS spectroscopic investigations. Based on the spectroscopic analysis, the structures shown in Figure 1 were proposed for compounds **1**–**4**. These structural conclusions were further confirmed by the close similarity (small deviations in the ^1^H- and ^13^C-NMR assignments are accounted for by the different solvents used for sample preparation) found between the collected spectroscopic data (see Supplementary Materials) and those reported in the literature for the proposed structures [13,14,15]. Although the absolute stereochemistry has not been determined in any cases, we assume that similar metabolic pathways led to the formation of the compounds in the fruiting bodies of *T. atrotomentosa* both in case of previous isolations and in the present study. Based on this, only the single enantiomers described in the literature are depicted in Figure 1.

Among the isolated metabolites there are two lactone type components: osmundalactone (**1**) and 5-hydroxy-hex-2-en-4-olide (**2**), while the other two, namely spiromentin C (**3**) and spiromentin B (**4**) possess a terphenylquinone skeleton. Osmundalactone (**1**) was obtained for the first time from this species in 1995 by Buchanan et al. [7]. Previously this compound has been already identified both in free form and as hydrolysis product of osmundalin (a glucoside of osmundalactone) in the fern species *Osmunda japonica* along with 5-hydroxy-hex-2-en-4-olide (**2**) [13,14]. Spiromentins B and C were identified in *T. atrotomentosa* by Besl et al., and indeed this species is the single source of these pigments described so far [4]. Spiromentines belongs to the group of terphenylquinones, which represent an interesting subclass of secondary metabolites. Natural occurrence of metabolites with a *p*-terphenyl core (also known as diphenylbenzenes or triphenyls) is essentially restricted to fungi and lichens. Several *p*-terphenylquinones with unusual structural features and notable biological properties (e.g., antiproliferative, antibacterial, antioxidant, and anti-inflammatory activity) have been described in the literature [15].

Compounds **1–4** were evaluated for their antimicrobial activity against 8 bacterial strains using the microdilution method. The isolated compounds proved to possess antimicrobial properties against both Gram-positive and -negative strains (Table 1).

Our experiments revealed that multiresistant *A. baumannii* and ESBL *E. coli* are the most susceptible against the studied compounds. *A. baumannii* and ESBL *E. coli* are human pathogens which have become resistant in many cases against the generally used antibacterial drugs causing serious nosocomial infections. Among the constituents 5-hydroxy-hex-2-en-4-olide (**2**) was the most active, although osmundalactone (**1**) and spiromentin C (**3**) have also shown significant effectiveness against these two resistant bacteria. The available data on the activity of mushroom extracts and their metabolites against *A. baumannii* are very scarce. In a study published in 2010 the activity of ethyl acetate extract of *Phellinus merrillii* against *A. baumannii* strains were reported to be fairly low with MIC values in the range of 0.71 and 1.42 mg/mL [16]. Schwan et al. screened more than 300 mushroom species native to North America for their potential activity against *A. baumannii*, but only three fungal species demonstrated more or less activity against this pathogen. Further chemical experiments identified the compound responsible for the observed antimicrobial activity, namely 2-aminoquinoline, isolated from *Leucopaxillus albissimus*. The MIC value of this metabolite determined for *A. baumannii* was 128 µg/mL [17]. In comparison the most effective compound in our experiments, 5-hydroxy-hex-2-en-4-olide (**2**) was found to have a MIC value of 6 µg/mL against *A. baumannii*.

In a previous study atromentin, a precursor molecule of the biosynthesis of other spiromentins, demonstrated antibacterial activity with MIC values in the range of 25–100 μg/mL [18]. The results of another study by Brewer et al. suggest that the antibiotic activity depends on the *para* substituent of the benzoquinone ring. The activity against *Bacillus subtilis* was found to be higher for 4,4-dimethoxyatromentin (MIC value of 5 μg/mL) with respect to atromentin (MIC value of 500 μg/mL) [19]. A similar tentative conclusion can be drawn for spiromentins by comparing the antimicrobial activities of the isomeric compounds, **3** and **4**. The almost complete loss of antibacterial activity parallel to the “replacement” of the furanyl with a pyranyl ring on the spiro carbon suggests that the antimicrobial activity depends on the substituents on the spiro carbon as well.

Compounds **1**–**4** were investigated with the aim of evaluating their synergistic effect with cefuroxime against MRSA using checkerboard techniques, though our results indicate that they do not enhance the activity of the studied antibiotic drug.

The isolated metabolites **1**–**4** were evaluated for their antioxidant activity using DPPH and ORAC assays. In the ORAC study spiromentins C (**3**) and B (**4**), displayed remarkable antioxidant effects (16.21 ± 0.38 and 11.23 ± 0.58 mmol TE/g, respectively), which were higher than the activity of ascorbic acid used as reference compound (6.97 ± 0.01 mmol TE/g) (Table 2).

Although osmundalactone (**1**) and 5-hydroxy-hex-2-en-4-olide (**2**) were less active, they are still considered compounds with notable antioxidant property. The compounds were also tested in the DPPH assay, but either did not show any activity (compound **1** and **2**) or could not be evaluated due to their color (purple) interference with the applied reagent at 550 nm.

In conclusion our results demonstrates that *T. atrotomentosa* is not only a species synthesizing a great variety of secondary metabolites, but also one of the very few mushroom sources of natural compounds with significant activity against multiresistant *A. baumannii* and ESBL *E. coli*. In this vein further detailed studies are warranted to explore the potential antibacterial activity of other mushroom species to identify novel anti-infective agents effective against resistant bacterial strains.

## 3. Materials and Methods

The chemicals used in the experiments were supplied by Sigma-Aldrich Hungary (Budapest, Hungary) and Molar Chemicals (Budapest, Hungary). Flash chromatography was carried out on a CombiFlash®Rf+Lumen instrument with integrated UV, UV-VIS and ELS detection using RediSep Rf Gold Normal Phase Silica Flash columns (4, 12 and 60 g) (Teledyne Isco, Lincoln, NE, USA). Normal-phase HPLC (NP-HPLC) separations were carried out on a Wufeng LC-100 Plus HPLC instrument equipped with a UV-VIS detector (Shanghai Wufeng Scientific Instruments Co., Ltd., Shanghai, China) at 254 nm, using a Zorbax-Sil column (250 × 4 mm, 5 μm; Agilent Technologies, Santa Clara, CA, USA). HRMS analyses were performed on an LTQ FT Ultra system (Thermo Fisher Scientific, Bremen, Germany). The samples were dissolved in methanol. The ionization method was ESI operated in positive ion mode. The protonated molecular ion peaks were fragmented by CID. Data acquisition and analysis were accomplished with Xcalibur software version 2.0 (Thermo Fisher Scientific, Waltham, MA, USA). NMR spectra were recorded at 25 °C on a Varian 500 or 800 MHz spectrometer Varian, Inc., Palo Alto, CA, USA) both equipped with a ^13^C sensitivity enhanced salt tolerant ^1^H/^13^C/^15^N cryogenically cooled probe head. Samples were dissolved in deuterated chlorofom or methanol-*d_4_* (Eurisotop, Saint-Aubin Cedex, France). Standard one and two dimensional pulse sequences, available in the VNMJ 3.2 library were used in all cases. Chemical shifts are reported in the delta scale using TMS (^1^H in the case of CDCl_3_), residual solvent signal (3.31/49.15 ppm for ^1^H/^13^C in the case of MeOD-*d4* or 77.0 ppm for ^13^C in case of CDCl_3_ as solvent) as references.

### 3.1. Mushroom Material

Sporocarps of *T. atrotomentosa* (2 kg) were collected in the vicinity of Szeged (Hungary) in August–October 2015. Fruiting bodies of *T. atrotomentosa* were stored at −20 °C until processing. A voucher specimen (No. P7) has been deposited at the Department of Pharmacognosy, University of Szeged, Hungary.

### 3.2. Extraction and Isolation

The fruiting bodies of *T. atrotomentosa* (2 kg) were extracted with methanol (11.5 L). After concentration, the dry methanol extract (90.0 g) was dissolved in 50% aqueous MeOH (600 mL) and solvent-solvent partition was performed with *n*-hexane and chloroform (5 × 500 mL each) yielding *n*-hexane, chloroform and aqueous MeOH-soluble phases. The chloroform-soluble phase was evaporated and the residue (8.56 g) was roughly separated with flash column chromatography on silica gel column using gradient system of *n*-hexane–acetone. The obtained fractions were combined based on TLC checking resulting in seven fractions (I–VII). Fraction II (882.5 mg) eluted with *n*-hexane–acetone 85:15, was further separated by multiple flash chromatography with increasing polarity of *n*-hexane–acetone. Two of the resulted fractions were finally purified with normal-phase HPLC using cyclohexane–isopropanol–water isocratic eluent system to result in compounds **1** (21.2 mg) and **2** (14.0 mg). Fraction IV (1.3 g) was subjected to flash column chromatography using *n*-hexane–acetone gradient system as mobile phase, and resulted in eight fractions (IV/1–8). The purification of IV/5 (63.4 mg) was performed by HPLC using mobile phase of cyclohexane–isopropanol–water 78:22:0.1 and led to the isolation of **3** (15.8 mg). Finally, fraction IV/6 (38.3 mg) was analyzed with HPLC applying an isocratic mobile phase of cyclohexane–isopropanol–water 75:25:0.1 yielding **4** (1.3 mg).

### 3.3. Determination of MIC Values

The in vitro antibacterial activities were assayed using microdilution method based on the guideline of Clinical and Laboratory Standards Institute against bacterial strains [20] including *Escherichia coli* (SZMC 6271), *Pseudomonas aeruginosa* (SZMC 2329), *Staphylococcus epidermidis* (SZMC 14531) *Staphylococcus aureus* (SZMC 14611), *Bacillus subtilis* (SZMC 0209), *Acinetobacter baumanii* (SZMC 24075), *Escherichia coli* ESBL (SZMC 24090), *Moraxella catarrhalis* (ATCC 25238). The suspensions of each bacteria were prepared from overnight broth cultures cultivated in Luria-Bertani broth (LB, 10 g/L tryptone, 10 g/L sodium chloride, 5 g/L yeast extract) at 37 °C and the concentrations of suspensions were adjusted to 10^5^ cells/mL. The DMSO solution of the investigated compounds as well as the reference agents were diluted with the LB media in the final concentration ranging from 1000 µg/mL to 6.25 µg/mL. The 96-well plates were prepared by dispensing into each well 100 μL of LB containing the bacterial cells and 100 μL of dissolved compounds and incubated for 24 h at 37 °C. The mixture of 100 μL LB broth and 100 μL sample solvent were used as blank sample for the background correction, while 100 μL of bacterial cultures with 100 μL solvents without the compounds was applied as positive control, while standard antibiotics were used as reference agents. Absorbance was measured at 620 nm after 24 h with microplate reader (SPECTROstar Nano, BMG Labtech, Ortenberg, Germany) and the MIC values were determined as the lowest concentration where the inhibition was higher than 10% of the positive control after the blank correction.

### 3.4. Investigation of Synergistic Effect Between Cefuroxime and the Isolated Ccompounds

Cefuroxime was used in checkerboard assay [21] in combination with the isolated compounds against MRSA (SZMC 6270). The suspensions of the bacteria were prepared from overnight broth cultures cultivated in LB broth at 37 °C and the concentrations of suspensions were adjusted to 10^5^ cells/mL. Into each well of the 96-well plates, 100 μL of LB broth were added containing the bacterial cells, which was completed with 50 μL of each compound and 50 μL of cefuroxime. Double dilutions of both the antibiotic and test compounds were carried out in five levels started from their related MIC values (isolated compounds: 250 μg/mL–15.6 μg/mL; cefuroxime: 300 μg/mL–18.75 μg/mL). The mixture of 100 μL LB broth and 100 μL sample solvents were used as blank sample for the background correction, while 100 μL of bacterial cultures with 100 μL solvents without the compounds and antibiotic was applied as positive control. The plates were incubated for 24 h at 37 °C and the absorbance of the wells were measured at 600 nm with microplate reader (SPECTROstar Nano) to observe for growth of the test organism, which was expressed in percentages of the positive control. The ∑FICIs were calculated as follows: ∑FICI = FIC A + FIC B, where FIC A is the MIC of drug A (cefuroxime) in the combination/MIC of drug A (cefuroxime) alone, and FIC B is the MIC of drug B (isolated compounds) in the combination/MIC of drug B (isolated compounds) alone. The combination is considered synergistic when the ∑FIC is ≤0.5, indifferent when the ∑FIC is >0.5 to <2, and antagonistic when the ∑FIC is ≥2.

### 3.5. Investigation of Antioxidant Activity

#### 3.5.1. DPPH Method

The analysis of free radical scavenging activity was carried out by the modified method of Miser-Salihoglu et al. [22]. The DPPH (2,2′-diphenyl-1-picrylhydrazyl) method was performed on a 96-well microplate. Microdilution series of extracts and the isolated compounds (concentration of 1 mg/mL dissolved in DMSO), were made starting from 150 µL. To each well 50 µL of DPPH solution (100 µM) was added. The absorbance was measured after 30 min at 550 nm with a FLUOstar Optima BMG Labtech plate-reader. The less active samples were measured again in 2 mg/mL concentration. Subsequent dilution series were made using the most effective samples, starting from 100 µL, to precisely evaluate half maximal effective concentration (EC_50_ values). As standard, ascorbic acid was used. DPPH and ascorbic acid standard were purchased from Sigma-Aldrich Hungary. EC_50_ (mg/mL) values were calculated using GraphPad Prism 6.0 software (GraphPad Software, La Jolla, CA, USA).

#### 3.5.2. ORAC Assay

The ORAC assay was carried out on a 96-well microplate according to the method of Mielnik et al. [23]. 20 µL of extracts or pure compounds of 0.01 mg/mL concentration were mixed with 60 µL of AAPH ((2,2′-azobis(2-methyl-propionamidine)dihydrochloride) (12 mM final concentration) and 120 µL of fluorescein solution (70 nM final concentrations), then the fluorescence was measured through 3 h with 1.5 min cycle intervals with FLUOStar Optima BMG Labtech plate-reader. All the experiments were carried out in triplicate, Trolox was used as standard. AAPH free radical and Trolox standard ((±)-6-hydroxy-2,5,7,8-tetramethyl-chromane-2-carboxylic acid) were purchased from Sigma-Aldrich Hungary. Fluorescein was purchased from Fluka Analytical (Tokyo, Japan). The antioxidant capacity was expressed as mmol Trolox equivalent per g of dry extract (mmol TEg^−1^), with help of GraphPad Prism 6.0 (GraphPad Software, La Jolla, CA, USA).

## Figures and Tables

**Table 1:** Antibacterial activity of compounds **1**–**4** expressed in MIC values.

Calculated MIC Values (µg mL^−1^)				
Compound	MACI	ESBL *E. coli*	*Mor. catarrhalis*	MRSA
**1**	10	10	–	250
**2**	6	10	50	250
**3**	20	10	50	250
**4**	–	100	–	–

MACI: multiresistant *Acinetobacter baumannii*, ESBL *E. coli*: extended-spectrum β-lactamase producing *Escherichia coli*, *Mor. catarrhalis*: *Moraxella catarrhalis*, MRSA: methicillin-resistant *Staphylococcus aureus*.

**Table 2:** Antioxidant activity of compounds **1**–**4** in ORAC assay.

Compound	ORAC Antioxidant Activity (mmol TE/g)
**1**	0.74 ± 0.30
**2**	3.85 ± 0.34
**3**	16.21 ± 0.38
**4**	11.23 ± 0.58
Ascorbic acid	6.97 ± 0.01

## Supplementary Materials

Supplementary materials are available online.
