# Supplementary material for: Bioactivity-Guided Isolation of Antimicrobial and Antioxidant Metabolites from the Mushroom Tapinella atrotomentosa
Source: Molecules. 2018 May 4;23(5):1082. doi: 10.3390/molecules23051082 (PMC6102582; doi:10.3390/molecules23051082)
Supplement: Supplementary file 1 [file molecules-23-01082-s001.pdf]

Supplementary materials

Bioactivity-guided isolation of antimicrobial and antioxidant metabolites from the mushroom *Tapinella atrotomentosa*

NMR spectra and spectral data on compound 1

^{1}H NMR (799.7 MHz, $CDCl_3$) δ: 6.85 (1H, dd, J = 9.9 Hz, J = 2.4 Hz, H-3), 5.99 (1H, dd, J = 9.9 Hz, J = 1.9 Hz, H-2), 4.38 (1H, dq, J = 8.8 Hz, J = 6.4 Hz, H-5), 4.21 - 4.29 (1H, m, H-4), 2.50 - 2.68 (1H, m, H-7), 1.49 (3H, d, J = 6.4 Hz, H-6)

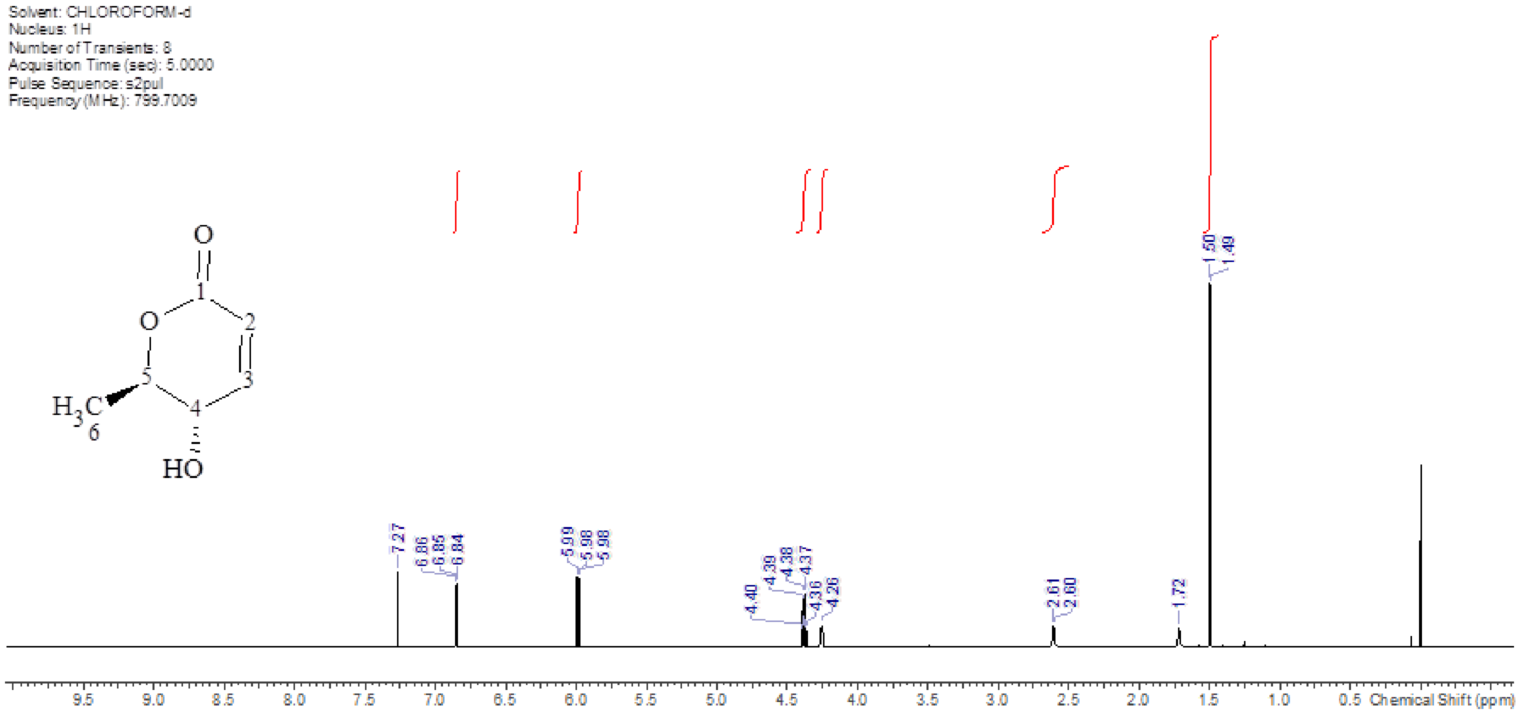

^{13}C NMR (201.1 MHz, $CDCl_3$) δ: 163.3 (C-1), 148.8 (C-3), 120.6 (C-2), 79.0 (C-5), 67.6 (C-4), 18.1 (C-6)

Solvent: CHLOROFORM-d
Nucleus: 13C
Number of Transients: 208
Acquisition Time (sec): 0.6554
Pulse Sequence: s2pul
Frequency (MHz): 201.1059

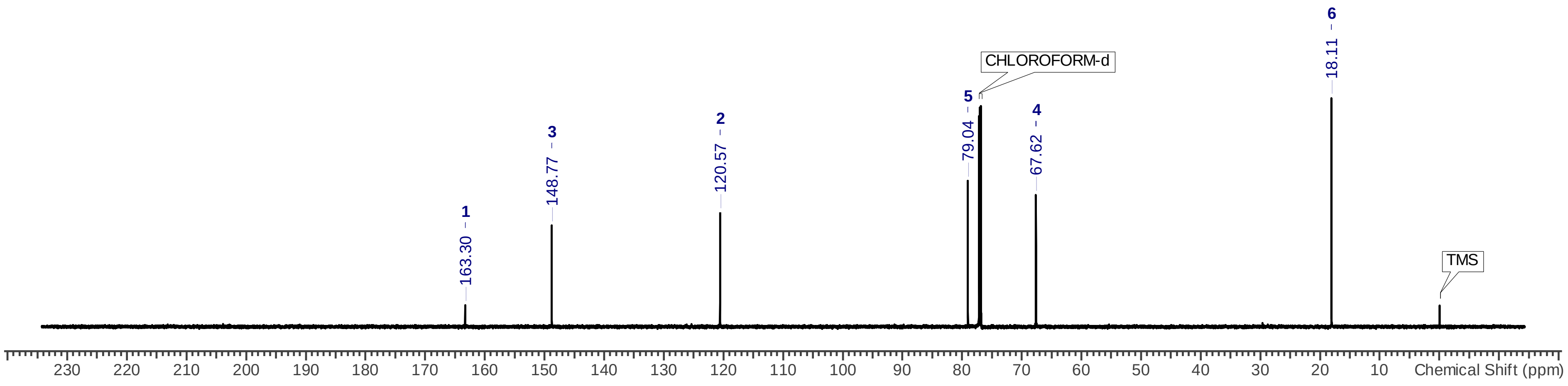

HRMS spectra and spectral data on compound 1

HRMS: M+H=129.05464 (delta=0.15 ppm; $C_6H_9O_3$). HR-ESI-MS-MS (CID=35%; rel. int. %): 111(2); 85(100).

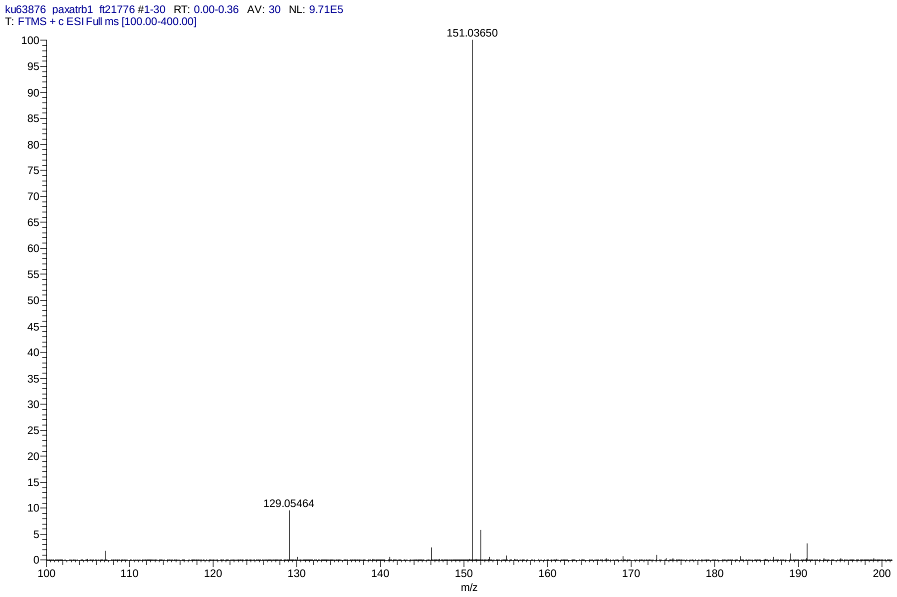

Full HRMS spectrum

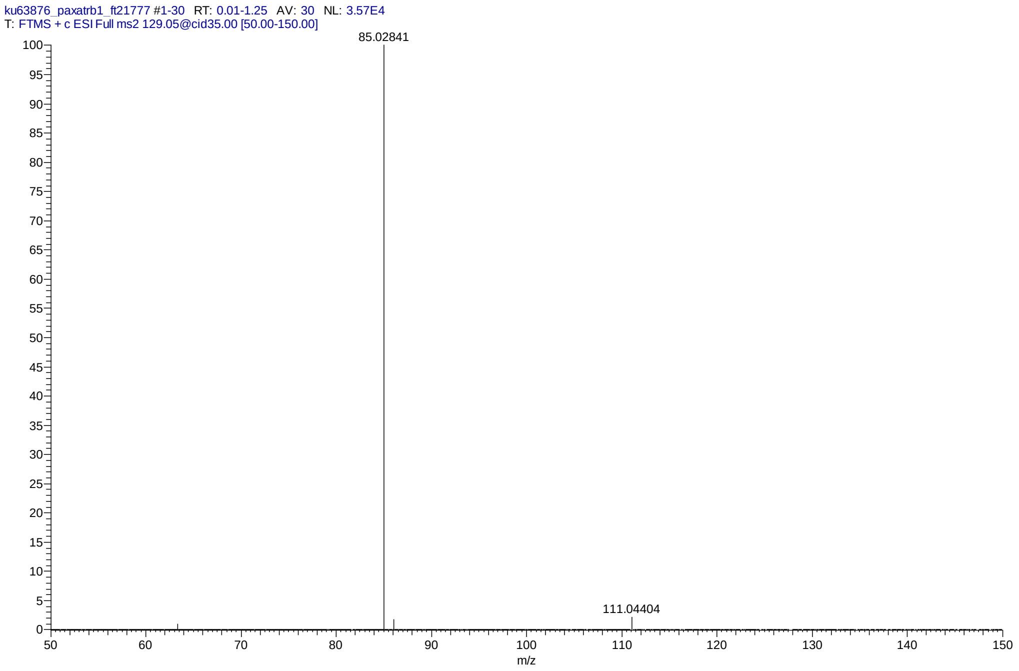

HR-MS/MS spectrum

NMR spectra and spectral data on compound 2

^{1}H NMR (799.7 MHz, $CDCl_3$) δ: 7.56 (1H, dd, J = 5.8 Hz, J = 1.6 Hz, H-3), 6.22 (1H, dd, J = 5.8 Hz, J = 2.1 Hz, H-2), 4.96 (1H, d, J = 4.7 Hz, H-4), 3.95 - 4.16 (1H, m, H-5), 1.33 (3H, d, J = 6.5 Hz, H-6)

Solvent: CHLOROFORM-d
Nucleus: 1H
Number of Transients: 1
Acquisition Time (sec): 5.0000
Pulse Sequence: s2pul
Frequency (MHz): 799.7009

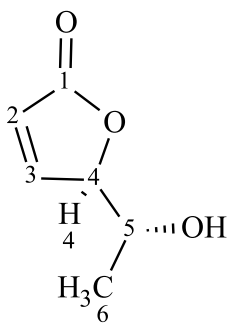

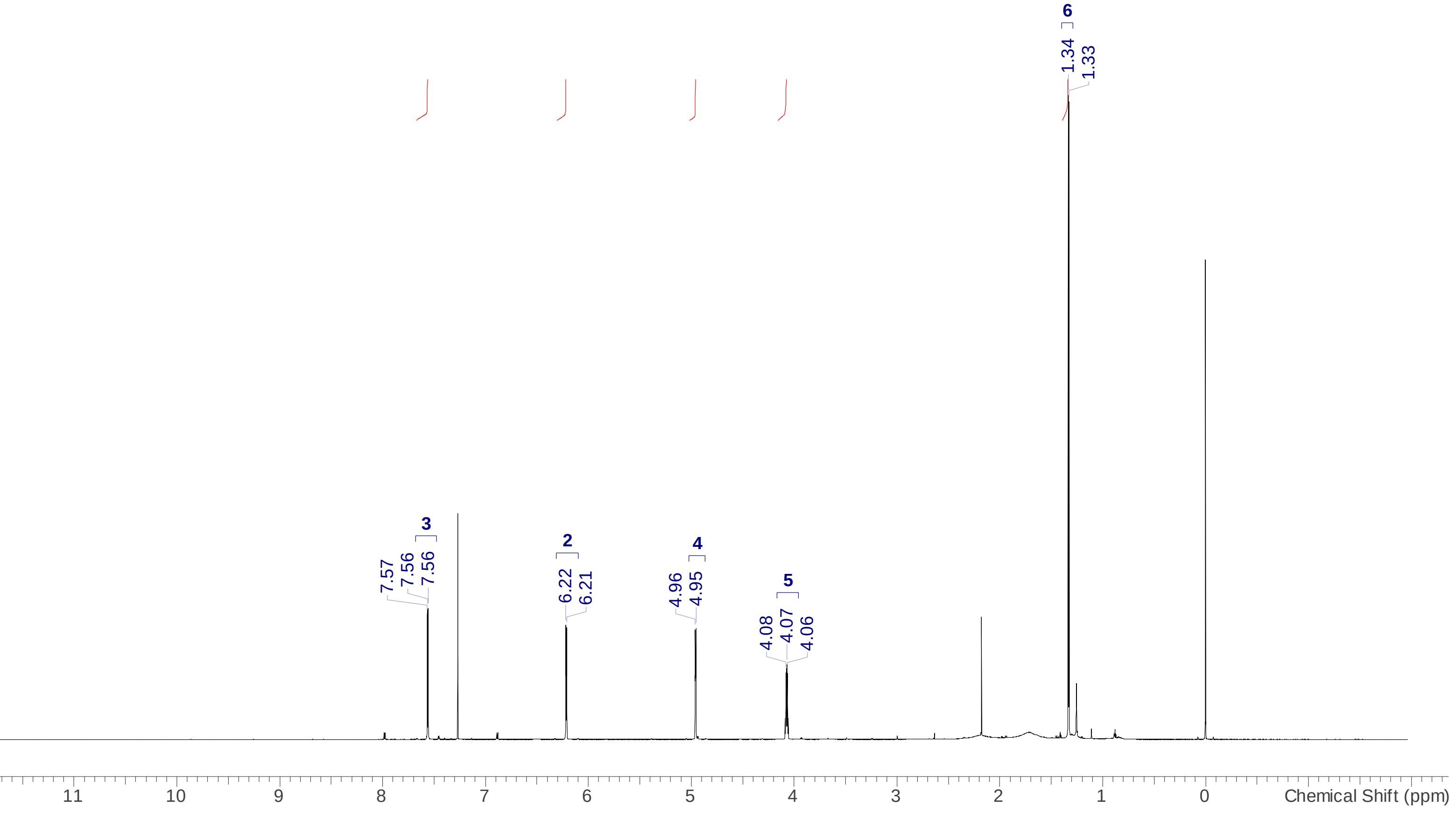

^{13}C NMR (201.1 MHz, $CDCl_3$) δ: 172.9 (C-1), 153.2 (C-3), 122.9 (C-2), 86.6 (C-4), 67.7 (C-5), 18.8 (C-6)

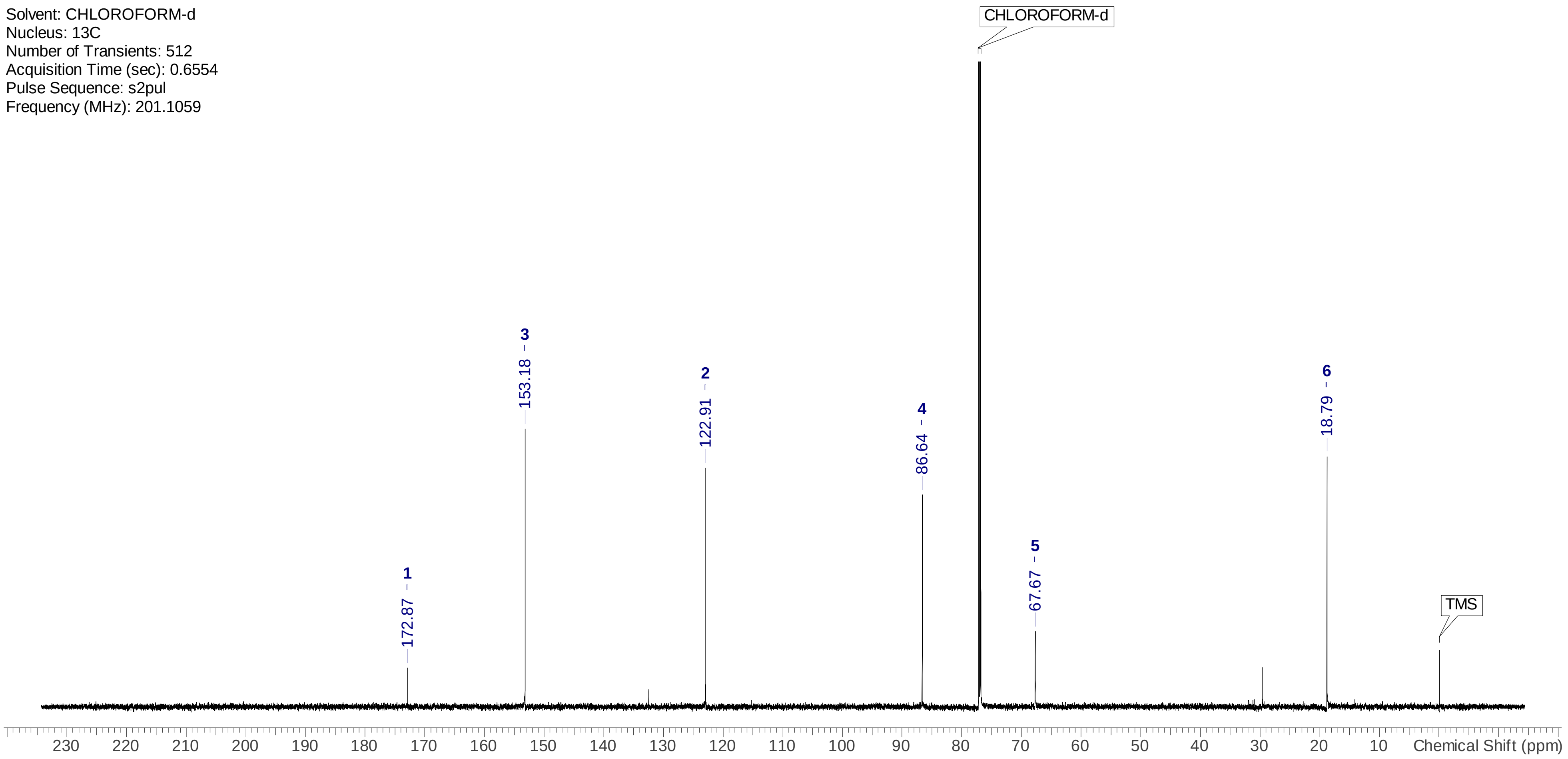

HRMS spectra and spectral data on compound 2

HRMS: M+H=129.05460 (delta=-0.16 ppm; $C_6H_9O_3$). HR-ESI-MS-MS (CID=35%; rel. int. %): 111(100); 85(83).

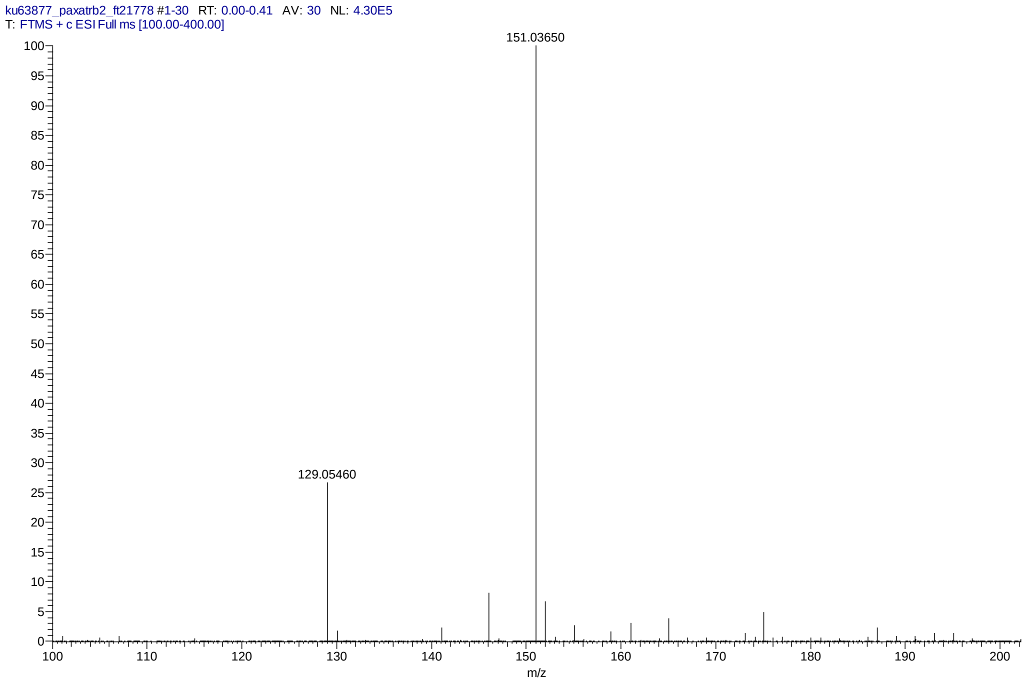

Full HRMS spectrum

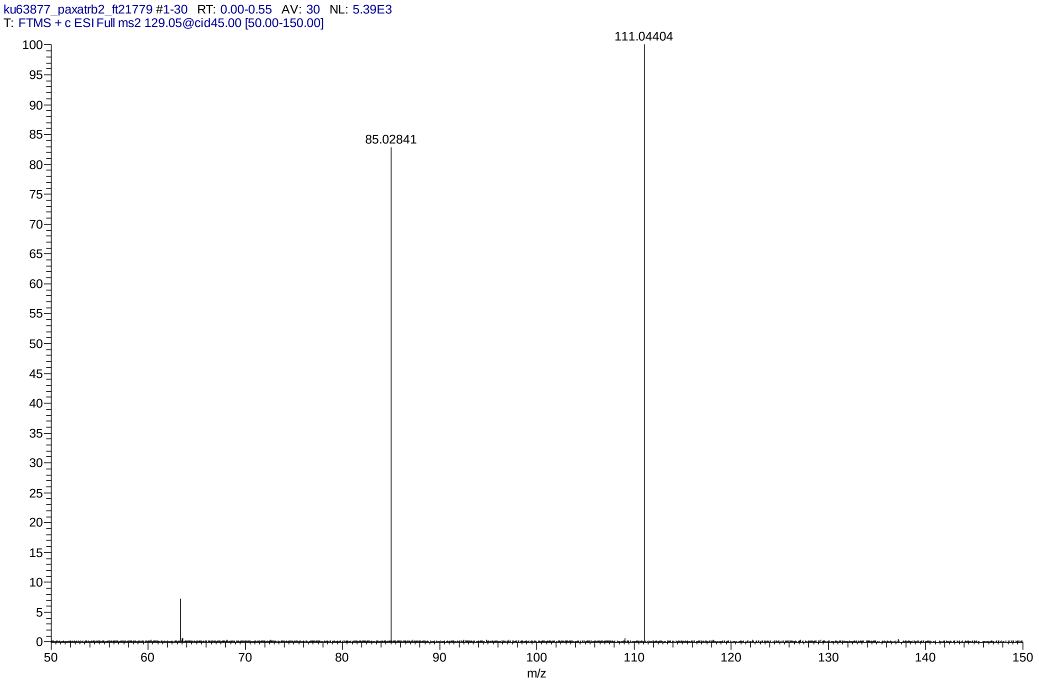

HR-MS/MS spectrum

NMR spectra and spectral data on compound 3

^{1}H NMR (499.9 MHz, CD_3OD) δ: 7.38 - 7.51 (4H, m, H-2'), 6.76 - 6.87 (4H, m, H-3'), 6.45 (1H, dd, J = 10.0 Hz, J = 1.5 Hz, H-5), 5.98 (1H, dd, J = 9.9 Hz, J = 2.0 Hz, H-6), 3.90 - 4.19 (2H, m, H-7, H-8), 1.41 (2H, d, J = 5.8 Hz, H-9)

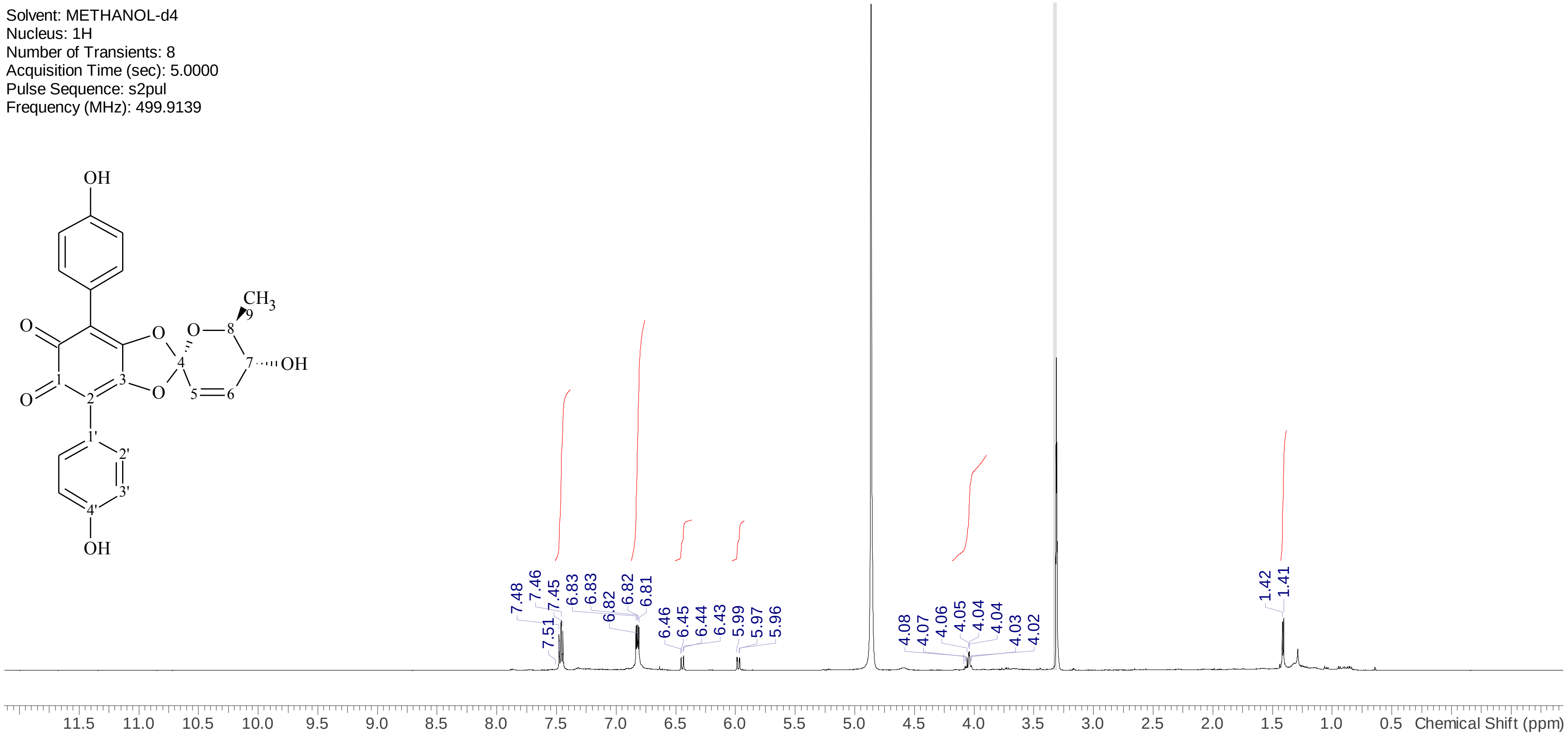

^{13}C NMR (125.7 MHz, CD_3OD) δ: 178.7/178.6 (C-1), 159.0/ 158.9 (C-4'), 157.3/156.9 (C-3), 143.0 (C-5), 132.41/132.35 (C-2'), 125.4 (C-4), 121.8/121.6 (C-1'), 121.5 (C-6), 116.03/116.00 (C-3'), 114.3/114.6 (C-2, 2), 78.5 (C-8), 68.3 (C-7), 18.1 (C-

9

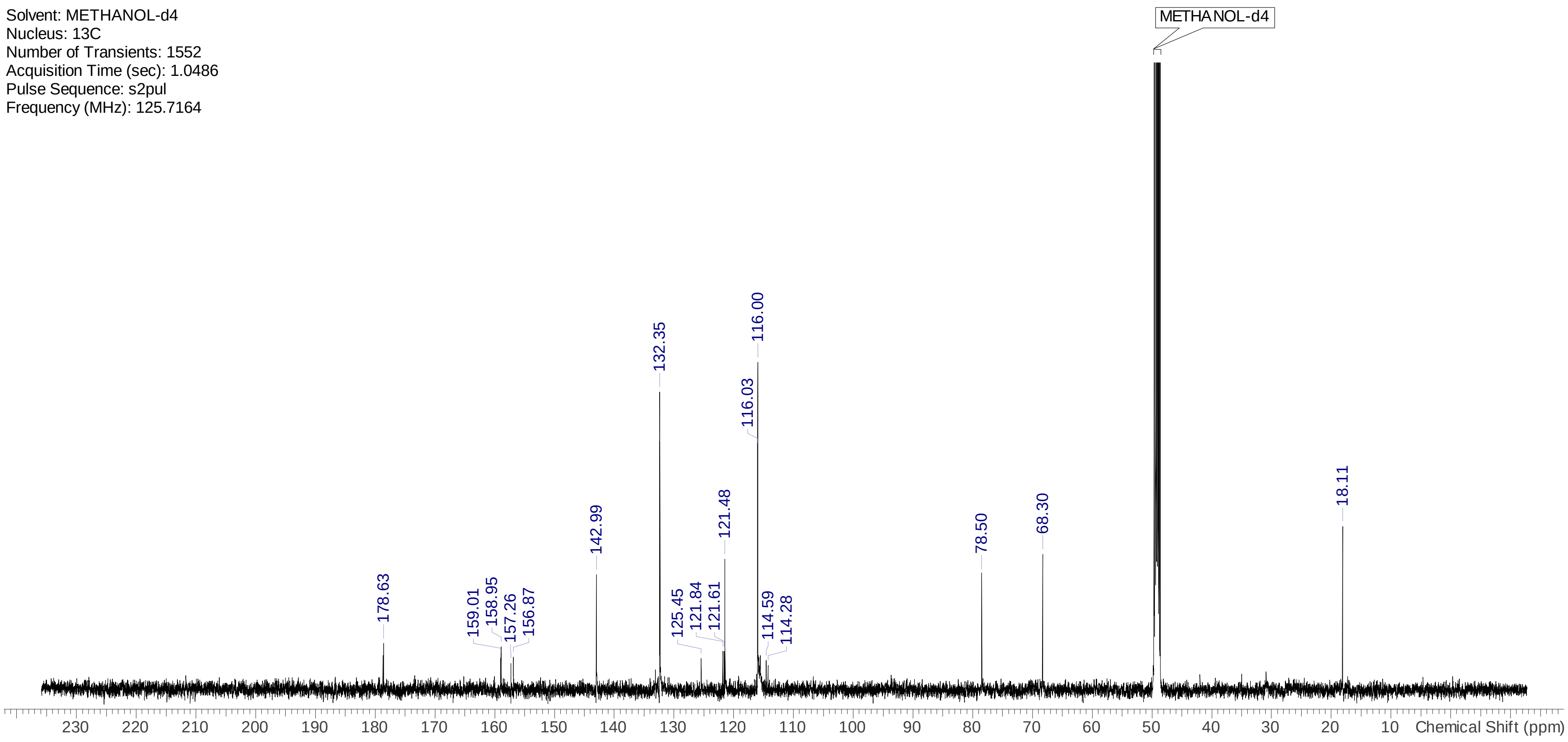

HRMS spectra and spectral data on compound 3

HRMS: M+H=435.10731 (delta=-0.31 ppm; $C_{24}H_{19}O_8$). HR-ESI-MS-MS (CID=35%; rel. int. %): 391(100); 325(6); 307(4).

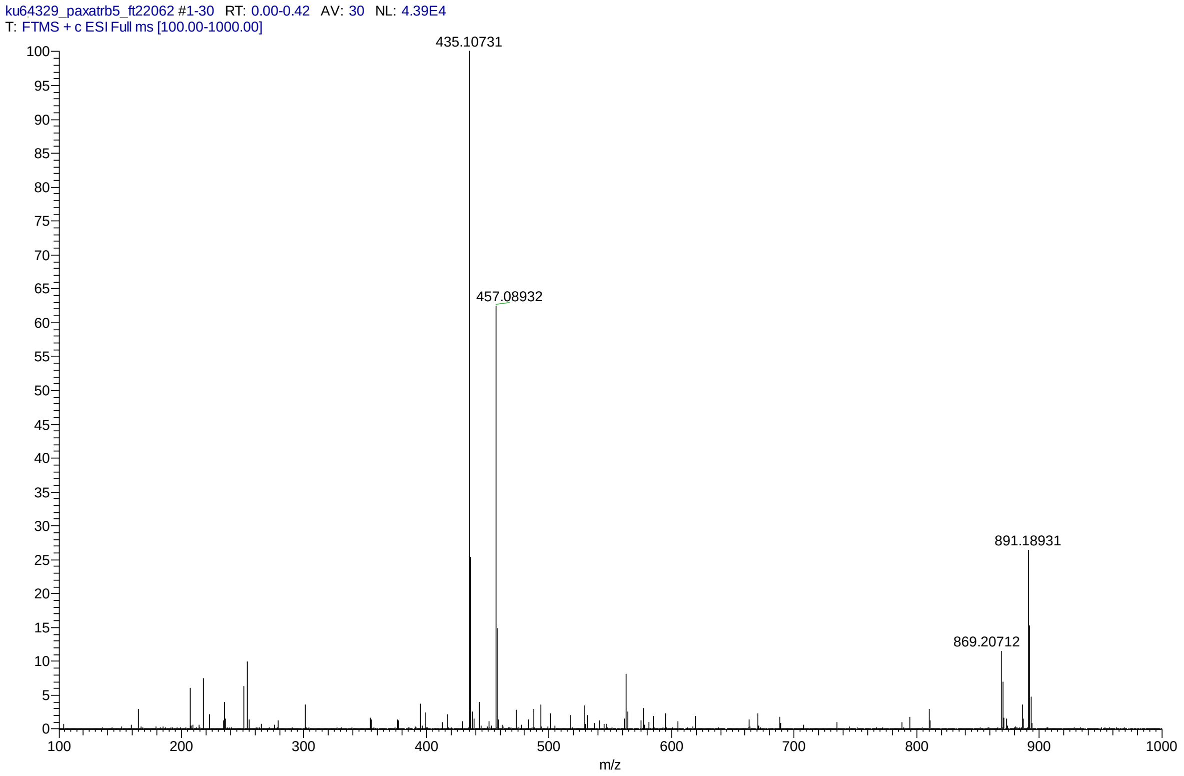

Full HRMS spectrum

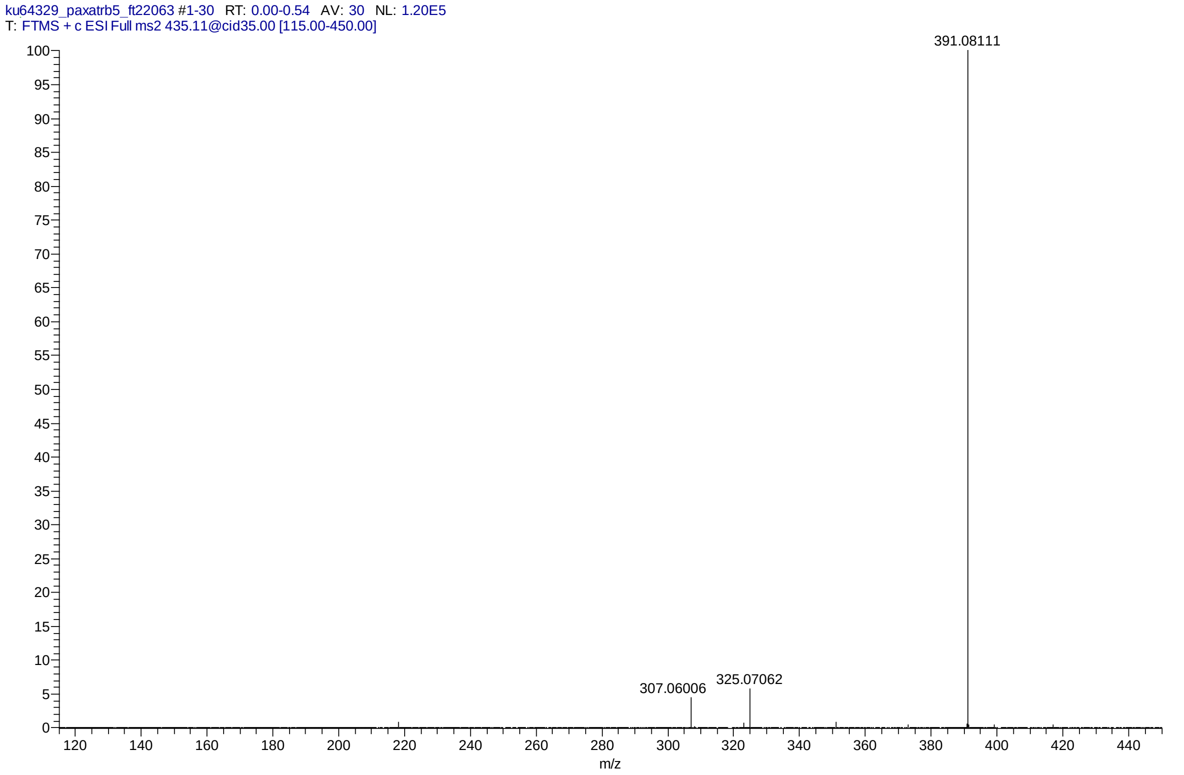

HR-MS/MS spectrum

NMR spectra and spectral data on compound 4

^{1}H NMR (499.9 MHz, CD_3OD) δ: 7.48 - 7.52 / 7.43 - 7.48 (4H, m, H-2'), 6.85 (1H, dd, J = 5.8 Hz, J = 1.4 Hz, H-6), 6.77 - 6.83 (4H, m, H-3'), 6.17 (1H, dd, J = 5.8 Hz, J = 2.2 Hz, H-5), 5.04 (1H, ddd, J = 4.8 Hz, J = 2.1 Hz, J = 1.4 Hz, H-7), 3.87 (1H, qd, J = 6.4 Hz, J = 4.8 Hz, H-8), 1.23 (3H, d, J = 6.4 Hz, H-9)

Solvent: METHANOL-d4
Nucleus: 1H
Number of Transients: 16
Acquisition Time (sec): 5.0000
Pulse Sequence: s2pul
Frequency (MHz): 499.9139

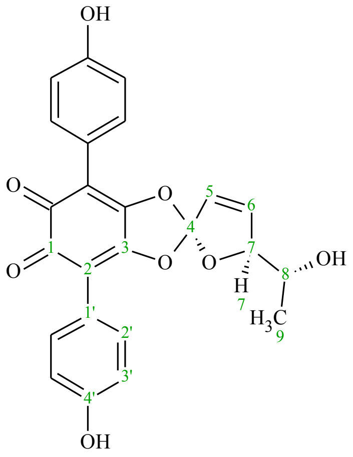

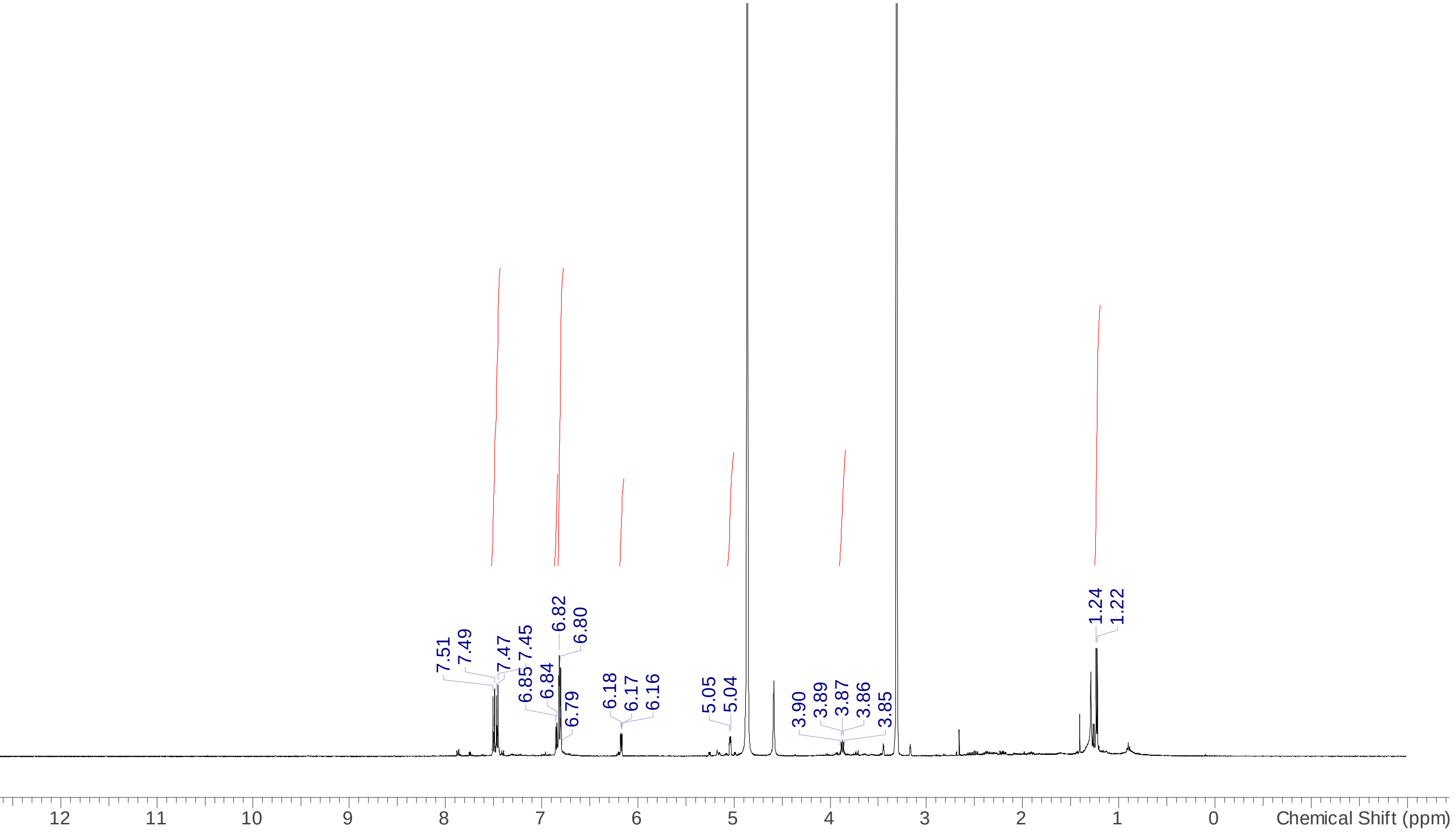

^{13}C NMR (125.7 MHz, CD_3OD) δ: 178.9/178.8 (C-1), 158.9/158.5 (C-4'), 153.7 (C-3), 140.9 (C-6), 139.3 (C-4)*, 132.40/132.37 (C-2'), 123.8 (C-5), 121.9 (C-1')*, 116.0 (C-3')*, 113.8 (C-2), 93.2 (C-7), 69.2 (C-8), 18.8 (C-9) *: determined from HMBC spectrum

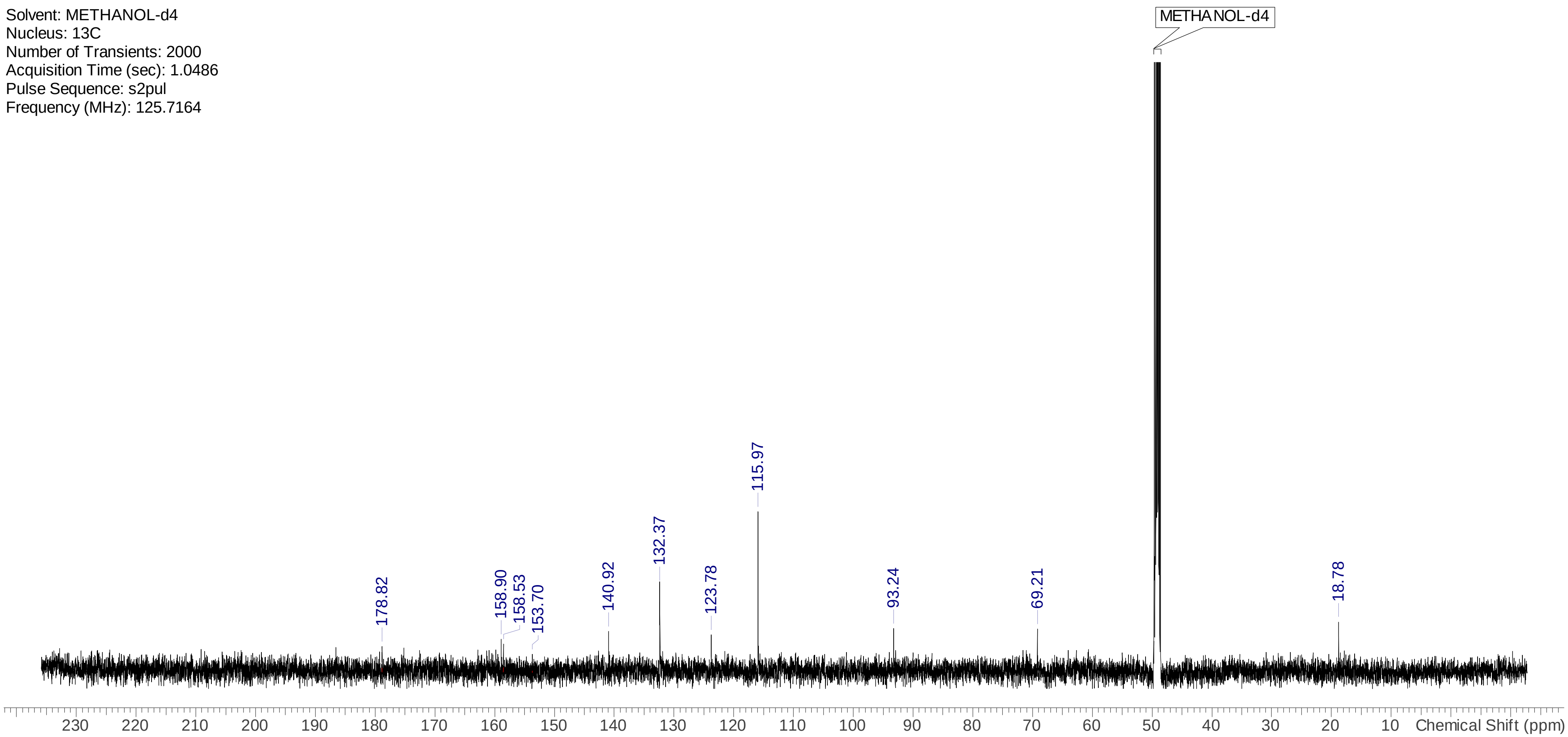

HRMS spectraand spectral data on compound 4

HRMS: M+Na=457.08913 (delta=-0.57 ppm; $C_{24}H_{18}O_8Na$).

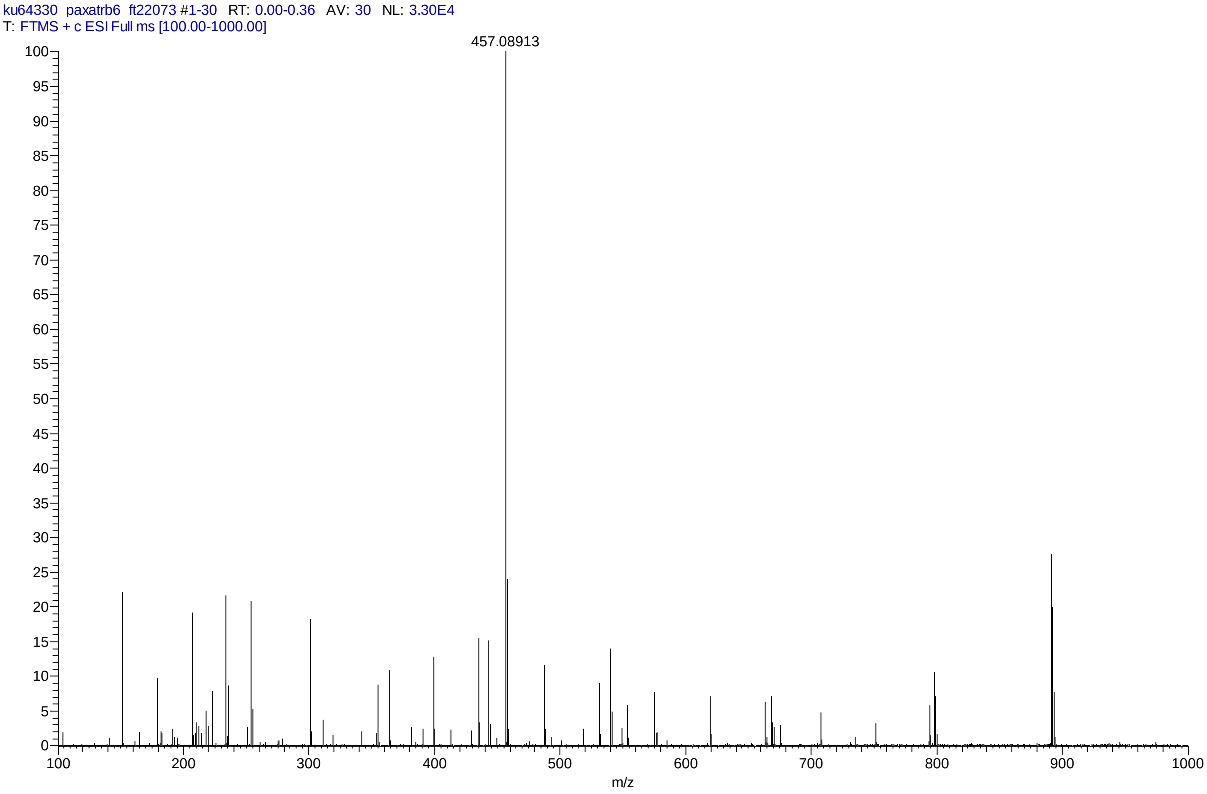

Full HRMS spectrum
